# Supplementary material for: Metabolomic Profiles of Mouse Tissues Reveal an Interplay between Aging and Energy Metabolism
Source: Metabolites. 2021 Dec 26;12(1):17. doi: 10.3390/metabo12010017 (PMC8779655; doi:10.3390/metabo12010017)
Supplement: Supplementary file 1 [file metabolites-12-00017-s001.zip › metabolites-1521528-supplementary_QZ_TM.pdf]

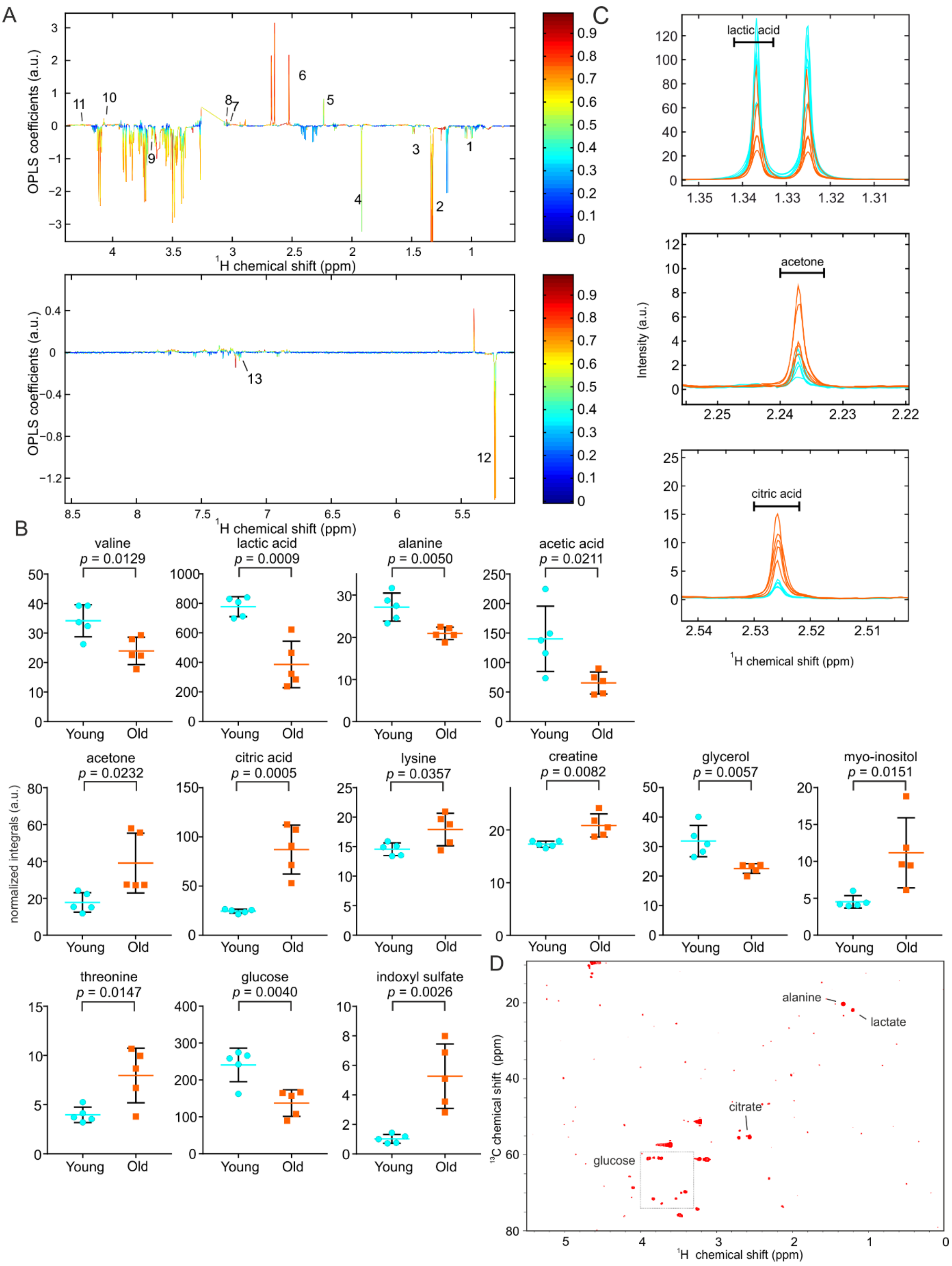

Supplementary Figure S1. (A) Reduced spectra based on normalized 1D ^{1}H NMR spectra of plasma samples showing metabolites significantly changed in levels. The numbers correspond to 1 valine, 2 lactic acid, 3 alanine, 4 acetic acid, 5 acetone, 6 citric acid, 7 lysine, 8 creatine, 9 glycerol, 10 myo-inositol, 11 threonine, 12 glucose, 13 indoxyl sulfate. (B) Plot of the quantifications of corresponding up- and down- regulated metabolites in (A), including means and standard deviations (SD) obtained from Student's t-test. All plotted metabolites are with $p < 0.05$. (C) Overlay of ^{1}H-NMR spectra of significantly up- and down- regulated metabolites. In each overlay, the 10 curves belong to the spectra

corresponding to the 10 plasma samples after normalization. Spectra of samples from young (9-10 weeks old) mice are shown in cyan, and from aged (96-104 weeks) mice are shown in orange. Areas delimitated by black bars are integrated areas, and correspond to selected metabolites shown in (B). (D) ^{1}H-^{13}C HSQC NMR spectrum for one plasma sample from a young mouse. Selected metabolites are annotated.

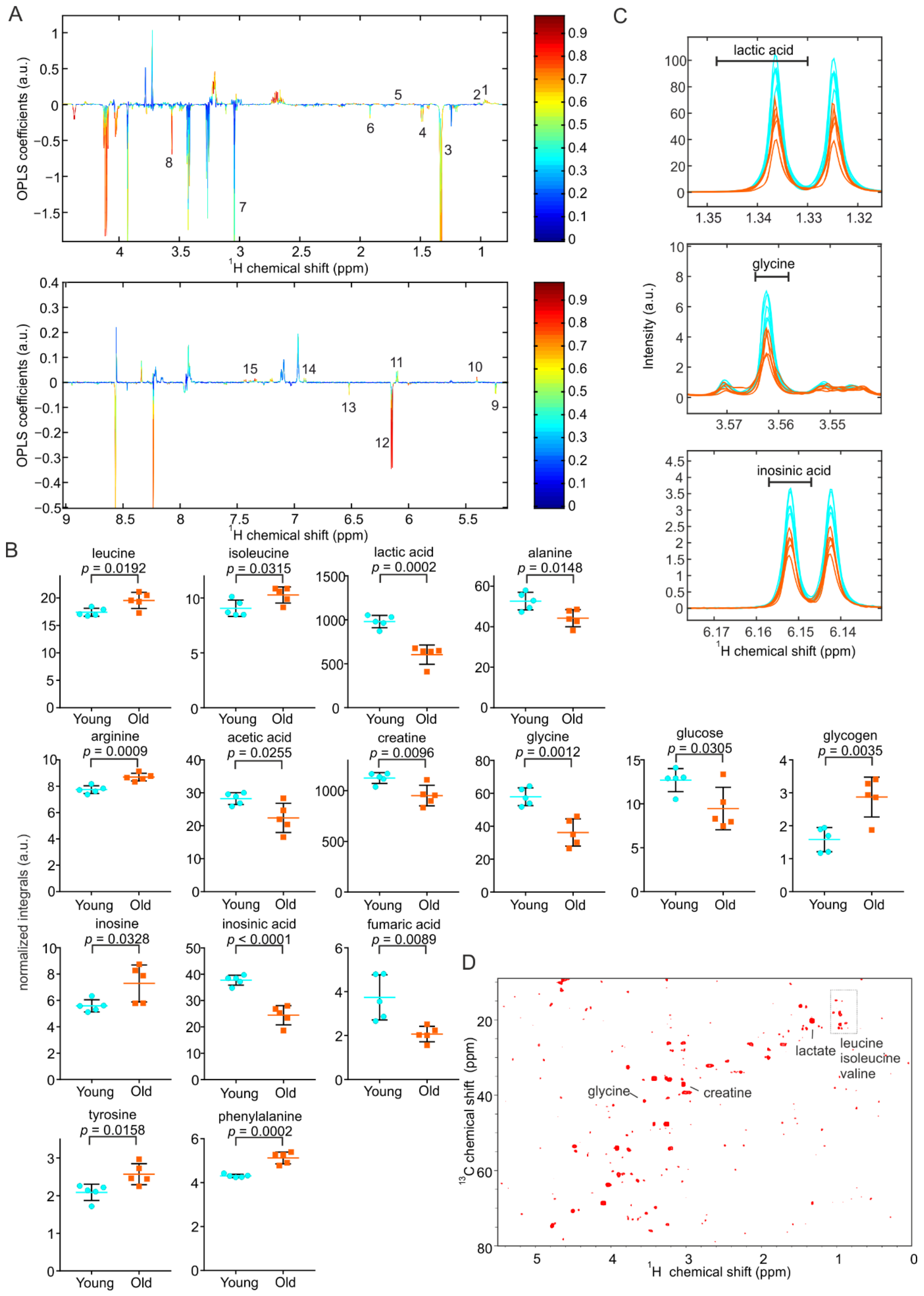

Supplementary Figure S2. (A) Reduced spectra based on normalized 1D ^{1}H NMR spectra of skeletal muscle samples showing metabolites significantly changed in levels. The numbers correspond to 1 leucine, 2 isoleucine, 3 lactic acid, 4 alanine, 5 arginine, 6 acetic acid, 7 creatine, 8 glycine, 9 glucose, 10 glycogen, 11 inosine, 12 inosinic acid, 13 fumaric acid, 14 tyrosine, 15 phenylalanine. (B) Plot of the

quantifications of corresponding up- and down- regulated metabolites in (A), including means and standard deviations (SD) obtained from Student's t-test. All plotted metabolites are with $p < 0.05$. (C) Overlay of ^{1}H-NMR spectra of significantly up- and down- regulated metabolites. In each overlay, the 10 curves belong to the spectra corresponding to the 10 skeletal muscle samples after normalization. Spectra of samples from young (9-10 weeks old) mice are in shown cyan, and from aged (96-104 weeks) mice are shown in orange. Areas delimitated by black bars are integrated areas, and correspond to selected metabolites shown in (B). (D) ^{1}H-^{13}C HSQC NMR spectrum for one plasma sample from an old mouse. Selected metabolites are annotated.

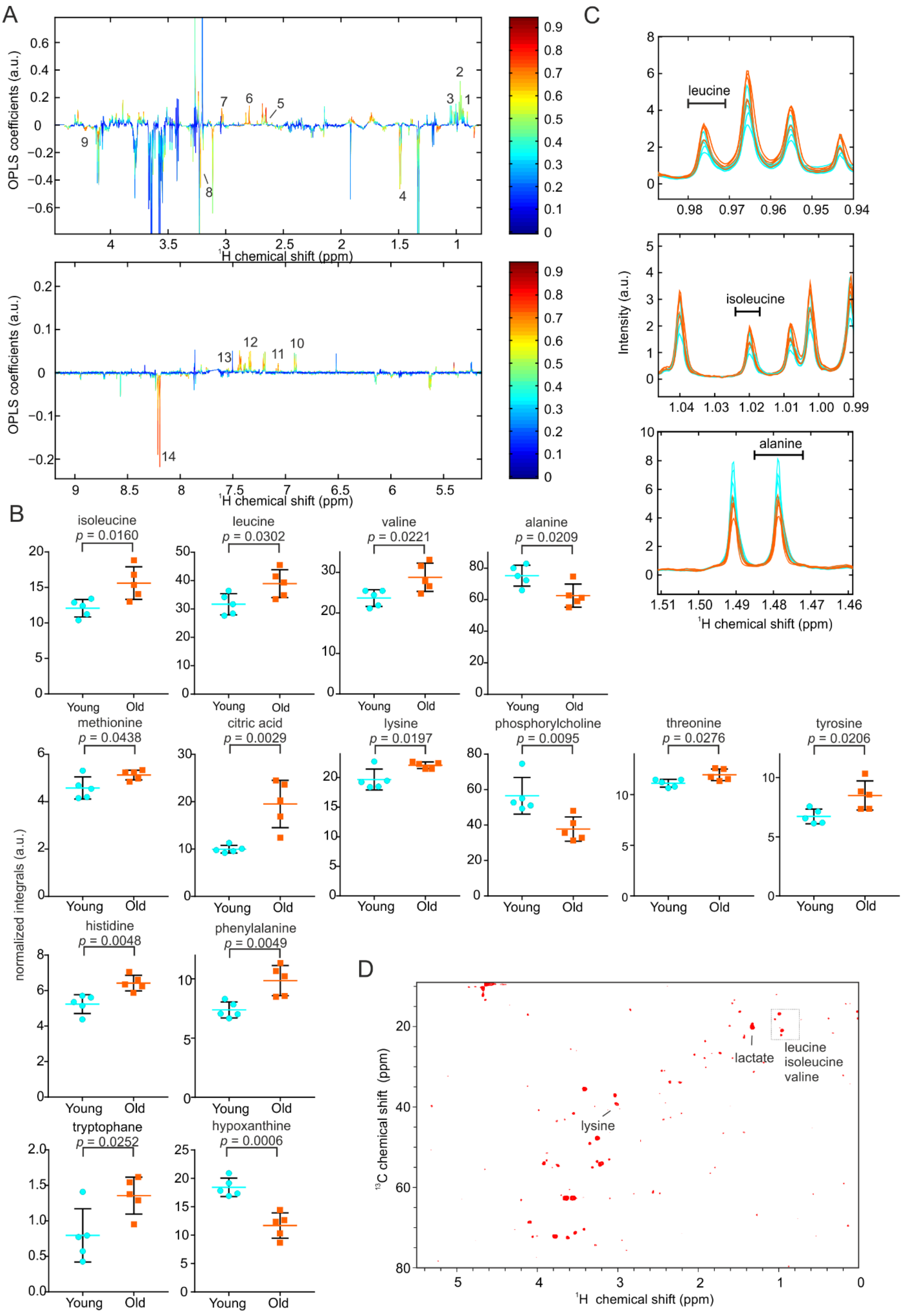

Supplementary Figure S3. (A) Reduced spectra based on normalized 1D ^{1}H NMR spectra of BAT samples showing metabolites significantly changed in levels. The numbers correspond to 1 isoleucine, 2 leucine, 3 valine, 4 alanine, 5 methionine, 6 citric acid, 7 lysine, 8 phosphorylcholine, 9 threonine, 10 tyrosine,

11 histidine, 12 phenylalanine, 13 tryptophane, 14 hypoxanthine. (B) Plot of the quantifications of corresponding up- and down- regulated metabolites in (A), including means and standard deviations (SD) obtained from Student's t-test. All plotted metabolites are with $p < 0.05$. (C) Overlay of ^{1}H-NMR spectra of significantly up- and down- regulated metabolites. In each overlap, the 10 curves belong to the spectra corresponding to the 10 BAT samples after normalization. Spectra of samples from young (9-10 weeks old) mice are shown in cyan, and from aged (96-104 weeks) mice are shown in orange. Areas delimitated by black bars are integrated area, and correspond to selected metabolites shown in (B). (D) ^{1}H-^{13}C HSQC NMR spectrum for one BAT sample from an old mouse. Selected metabolites are annotated.

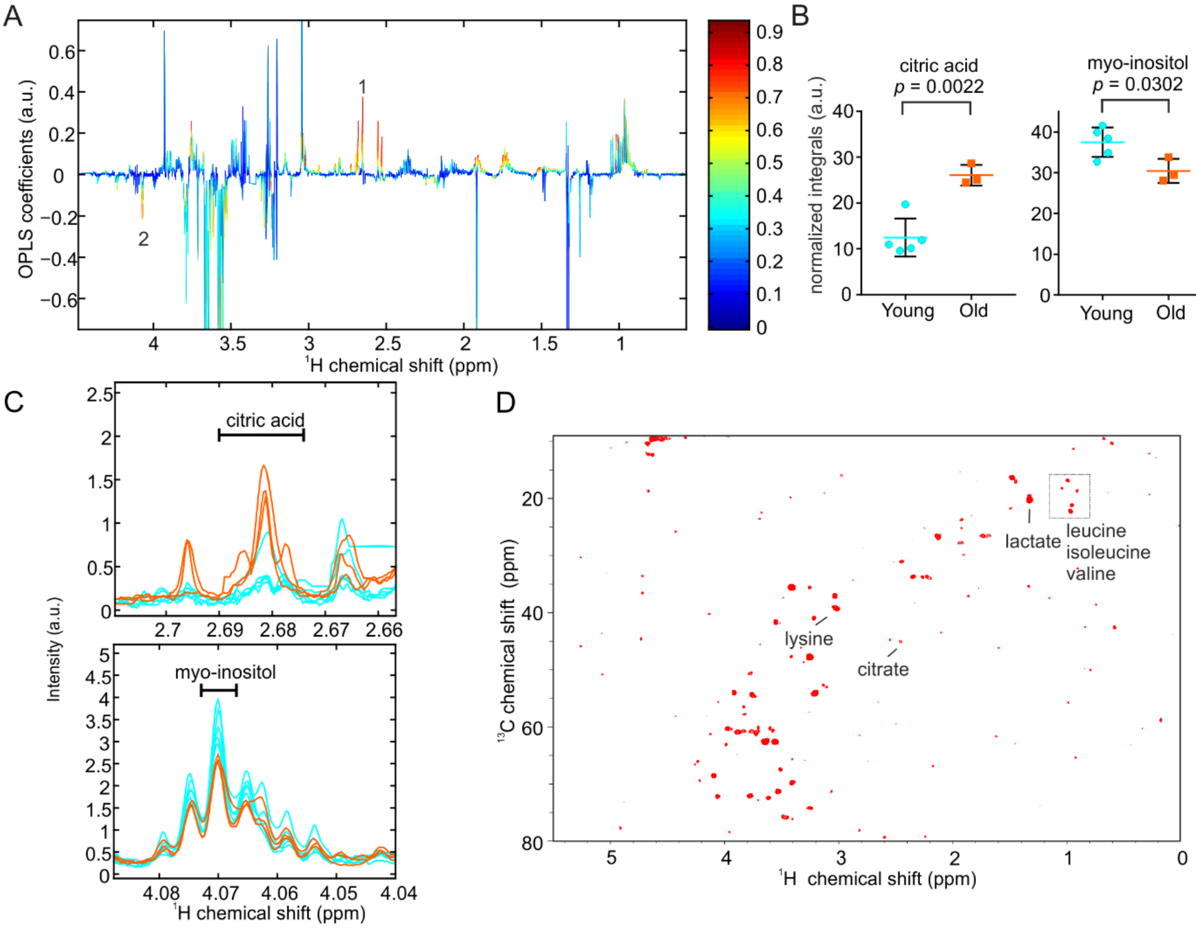

Supplementary Figure S4. (A) Reduced spectra based on normalized 1D ^{1}H NMR spectra of WAT samples showing metabolites significantly changed in levels. The numbers correspond to 1 citric acid, 2 myo-inositol. (B) Plot of the quantifications of corresponding up- and down- regulated metabolites in (A), including means and SDs obtained from Student's t-test. All plotted metabolites are with $p < 0.05$. (C) Overly of ^{1}H-NMR spectra of significantly up- and down- regulated metabolites. In each overlay, the 8 curves belong to the spectra corresponding to the 8 WAT samples after normalization. Spectra of samples from young (9-10 weeks old) mice are shown in cyan, and from aged (96-104 weeks) mice are shown in orange. Areas delimitated by black bars are integrated areas and correspond to metabolites shown in (B). (D) ^{1}H-^{13}C HSQC NMR spectrum for one WAT sample from a young mouse. Selected metabolites are annotated.

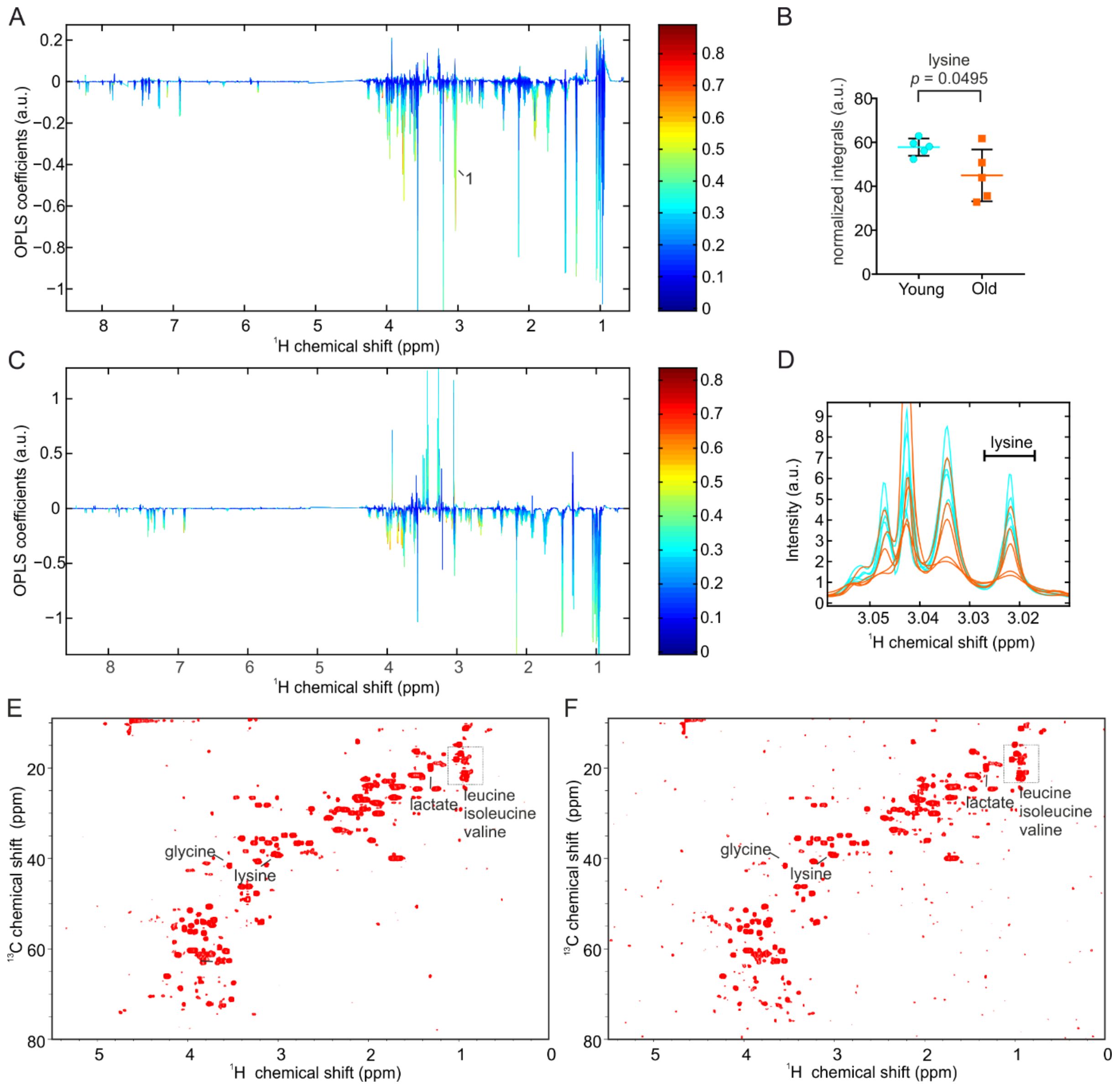

Supplementary Figure S5. (A) Reduced spectra based on normalized 1D ^{1}H NMR spectra of duodenum samples showing alteration of metabolites in levels. The numbers correspond to 1 lysine. (B) Plot of the quantifications of corresponding up- and down- regulated metabolites in (A), including means and SDs obtained from Student's t-test. All plotted metabolites are with $p < 0.05$. (C) Reduced spectra based on normalized 1D ^{1}H NMR spectra of jejunum samples showing alteration of metabolites in levels. (D) Overlay of ^{1}H-NMR spectra of lysine from duodenum. All 10 curves belong to the spectra corresponding to the 10 duodenum samples after normalization. Spectra of samples from young (9-10 weeks old) mice are shown in cyan, and from aged (96-104 weeks) mice are shown in orange. The area delimitated by a black bar indicates the integrated areas and correspond to lysine shown in (B). (E) ^{1}H-^{13}C HSQC NMR spectrum for one duodenum sample from an old mouse. (F) ^{1}H-^{13}C HSQC NMR spectrum for one jejunum sample from an old mouse. Selected metabolites are annotated.

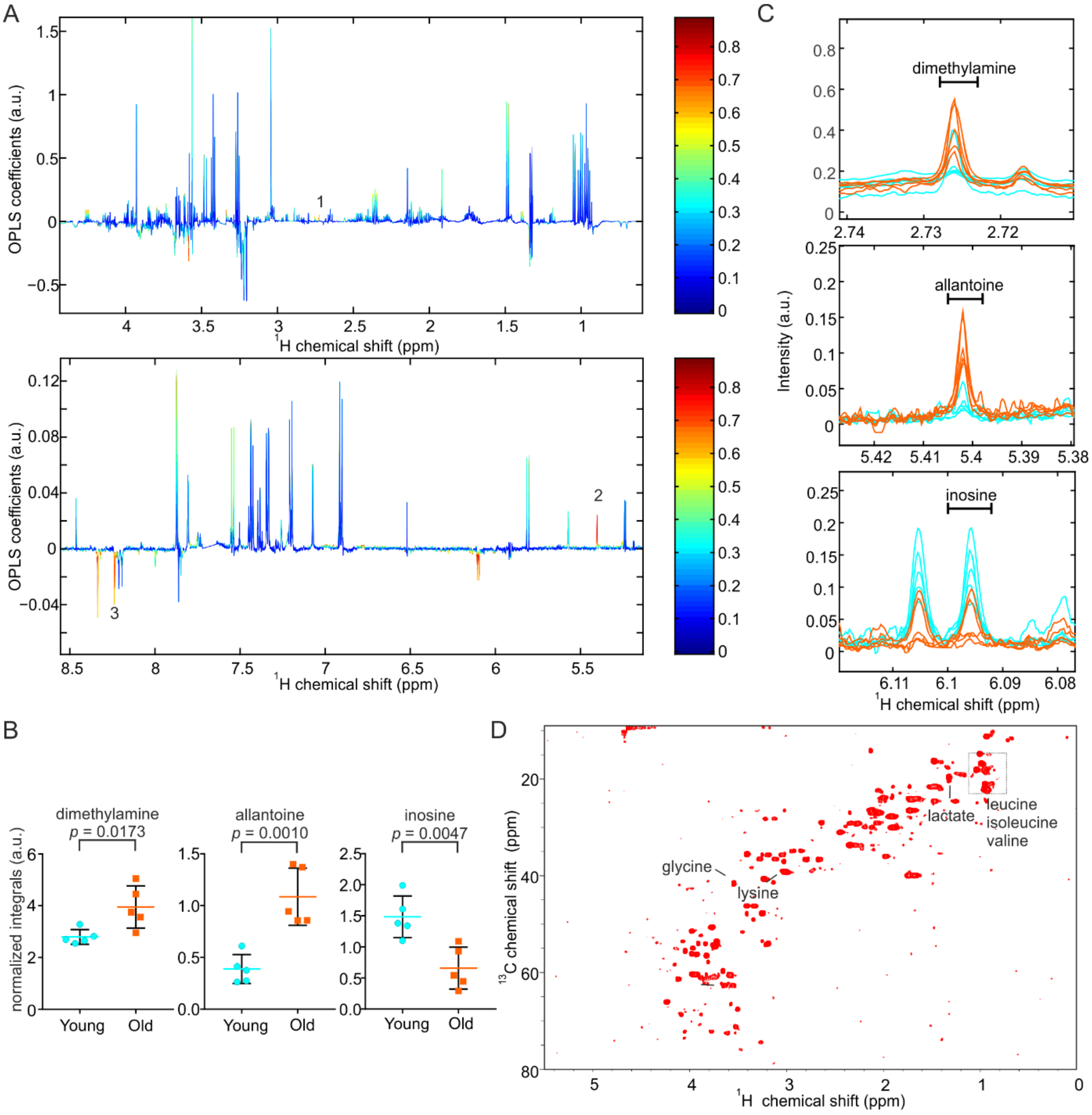

Supplementary Figure S6. (A) Reduced spectra based on normalized 1D ^{1}H NMR spectra of ileum samples showing alteration of metabolites in levels. The numbers correspond to 1 dimethylamine, 2 allantoine, 3 inosine. (B) Plot of the quantifications of corresponding up- and down- regulated metabolites in (A), including means and SDs obtained from Student's t-test. All plotted metabolites are with $p < 0.05$. (C) Overlay of ^{1}H-NMR spectra of significantly up- and down-regulated metabolites. In each overlay, the 10 curves belong to the spectra corresponding to the 10 ileum samples after normalization. Spectra of samples from young (9-10 weeks old) mice are colored in cyan, and from aged (96-104 weeks) mice are colored in orange. Areas delimitated by black bars are integrated areas, corresponding to metabolites shown in (B). (D) ^{1}H-^{13}C HSQC NMR spectrum for one ileum sample from an old mouse. Selected metabolites are annotated.
